# A Case of Strangulated Hernia

**Published:** 1875-01

**Authors:** J. W. Wimbish

**Affiliations:** Bonner, Lincoln Parish, Louisiana


### A CASE OF STRANGULATED HERNIA.

BY J. W. WIMBISH, M.D., OF BONNER, LINCOLN PARISH, LOUISIANA.

On the 30th day of July, of the present year, Nathan Duckworth, f. m. c., while going to a public speaking, stepped suddenly into a gully, when he found, to use his own language, that his bowels had come down. He undertook to put them back, with the assistance of several other freedmen who were present, but failed to do so. Dr. O. B. S., an old practitioner of the neighborhood, was sent for, who, upon making an examination, found the patient laboring under an oblique inquinal hernia. He attempted reduction by the taxis, but did not succeed (though it shouid be stated that the doctor had no chloroform with him, and consequently did not use any). I was sent for late in the evening, the accident having occurred about 10 o'clock A.M. I found the patient lying in an old field, near by where the accident happened. He was vomiting incessantly, bathed in a clammy perspiration, and apparently suffering the most intense pain. After manipulating to a very limited extent, the patient being slightly under the influence of chloroform, I concluded that relief could only be obtained by an operation, and suggested that he be moved to a house about a half mile distant. By request of the attending physician, I concluded to operate by torchlight, feeling confident that if he remained in his then present condition until morning, that the success of an operation would be greatly diminished. It being the first case of the kind that I have had an opportunity of operating upon during my practice, I was anxious for it to be a success, and equally cautious. The patient being placed under the influence of chloroform, I proceeded very carefully to divide the different layers of fasciæ (though failing to single each out, as I have heard Prof. Y. G. Richardson, of the University of Louisiana, say that he could not do himself); however, I reached the intestine, and running the point of my left index finger along the incision in the direction of the canal, found the constriction at the external ring, which I divided and effected a reduction. I ordered a dose of opium to be given every three hours, and went home. I visited the patient next morning and found him doing well. I did not see him any more, but heard that he went home in five days.

If you think that a publication of the foregoing case is calculated to impress the mind of any practitioner of medicine with the necessity of early interference in cases of strangulated hernia, you will please do so.

